# Behavior of magnetoelectric hysteresis and role of rare earth ions in multiferroicity in double perovskite Yb_2_CoMnO_6_

**DOI:** 10.1038/s41598-021-03330-8

**Published:** 2021-12-10

**Authors:** Jong Hyuk Kim, Ki Won Jeong, Dong Gun Oh, Hyun Jun Shin, Jae Min Hong, Jin Seok Kim, Jae Young Moon, Nara Lee, Young Jai Choi

**Affiliations:** Department of Physics, Yonsei University, Seoul, 03722 Korea

**Keywords:** Physics, Condensed-matter physics, Ferroelectrics and multiferroics

## Abstract

Double-perovskite multiferroics have been investigated because alternating orders of magnetic ions act as distinct magnetic origins for ferroelectricity. In Yb_2_CoMnO_6_, the frustrated antiferromagnetic order emerging at *T*_N_ = 52 K induces ferroelectric polarization perpendicular to the *c* axis through cooperative O^2−^ shifts via the symmetric exchange striction. In our detailed measurements of the magnetoelectric properties of single-crystalline Yb_2_CoMnO_6_, we observe full ferromagnetic-like hysteresis loops that are strongly coupled to the dielectric constant and ferroelectric polarization at various temperatures below *T*_N_. Unlike Lu_2_CoMnO_6_ with non-magnetic Lu^3+^ ions, we suggest the emergence of additional ferroelectric polarization along the *c* axis below the ordering temperature of magnetic Yb^3+^ ions, *T*_Yb_ ≈ 20 K, based on the spin structure established from recent neutron diffraction experiments. While the proposed description for additional ferroelectricity, ascribed to the symmetric exchange striction between Yb^3+^ and Co^2+^/Mn^4+^ magnetic moments, is clearly given, anomalies of dielectric constants along the *c* axis are solely observed. Our interesting findings on magnetoelectric hysteresis and the possible development of additional ferroelectricity reveal notable characteristics of double perovskites and provide essential guidance for the further examination of magnetoelectric functional properties.

## Introduction

Condensed-matter systems with strongly coupled order parameters offer immense opportunities for a fundamental understanding of their governing interactions as well as for the utilization of new technologies. Interesting cross-coupling effects can be observed in magnetoelectric and multiferroic materials, whose interlinked electric and magnetic order parameters have inspired materials research to explore new multifunctional materials and investigate the mechanisms underlying magnetoelectiricity^1–4^. Research has been aimed at discovering ferroelectricity driven by a specific type of ordered magnetic state. Ferroelectric distortions have been ascribed to both symmetric and antisymmetric parts of the magnetic exchange striction^5,6^. Because of this origin, the substantial coupling between structural distortions and magnetic order often results in a large variation in the dielectric and ferroelectric properties through the application of magnetic fields^2–4,7^. Although various magnetic materials have been known to be magnetoelectrics or multiferroics, exploring new materials with cross-couplings is still beneficial for improving the feasibility of multiple functionalities.

Double-perovskite compounds, in which two different transition-metal ions are alternately located in octahedral environments, have been widely investigated. In such materials, combinations of mixed-valence magnetic ions reveal the intricate magnetic interactions and ionic valence/antisite disorders, which enable various fascinating physical properties, such as metamagnetism^8–10^, exchange bias^11–13^, magnetocaloric effect^14–17^, and multiferroicity^18–21^. Recent studies have also focused on the potential use of double-perovskite halides in energy devices such as photovoltaic devices^22–24^, photocatalysts^25–28^, UV detectors^29,30^, and solar energy storage^31,32^. Double-perovskite R_2_CoMnO_6_ (R = La, …, Lu) compounds crystallize in a monoclinic double-perovskite structure (*P2*_*1*_/*n* space group), in which alternating Co^2+^ and Mn^4+^ ions are located in corner-shared octahedral O^2−^ environments^33^. Co^2+^ and Mn^4+^ superexchange interactions result in a long-range ferromagnetic order, whose ordering temperature varies from 204 K for La_2_CoMnO_6_ to 67 K for Er_2_CoMnO_6_ as the size of the rare-earth ions decreases^33^. It has been known that the incomplete alteration of the Co^2+^ and Mn^4+^ ions results in a small portion of additional antiferromagnetic clusters with Co^2+^- Co^2+^ or Mn^4+^- Mn^4+^ pairs, which generate anti-sites of ionic disorders and/or antiphase boundaries^34–37^. Another valence state of Co^3+^- Mn^3+^ can be additionally formed as antiferromagnetic clusters^38^. In Yb_2_CoMnO_6_ (YCMO) and Lu_2_CoMnO_6_, significant distortions of O^2−^ octrahedra owing to the smaller size of rare-earth ions induce a magnetic frustration associated with the nearest-neighbor ferromagnetic and next-nearest-neighbor antiferromagnetic exchange interactions^39^. These frustrated interactions lead to up-up-down-down (↑↑↓↓) spin ordering along the *c* axis with ordering temperatures of 52 and 48 K for YCMO and Lu_2_CoMnO_6_, respectively^40^.

Lu_2_CoMnO_6_ is a double-perovskite multiferroic; it is a rare example of a multiferroic that exhibits ferromagnetic-like magnetic hysteresis with net magnetization and strong coupling to the dielectric constant and ferroelectric polarization in response to external magnetic fields^20,41^. The ↑↑↓↓ spin arrangement formed by frustrated exchange interactions has been known to generate ferroelectric polarization perpendicular to the *c* axis through the cooperative O^2−^ shifts via the symmetric exchange striction^20,21,42^. In Er_2_CoMnO_6_, the ferrimagnetic order activated by Er^3+^ moments antiparallelly aligned with ferromagnetic Co^2+^/Mn^4+^ sublattices results in an inversion of the magnetic hysteresis loop^9,43^. Moreover, the additional small portion of multiferroic phase coexisting with the ferrimagnetic phase has been presented as simultaneous metamagnetic and ferroelectric transitions^41^. Despite the clear demonstrations of correlated magnetic and ferroelectric properties in such multiferroics, the full hysteretic behavior in coupled magnetization and polarization and the role of magnetic rare-earth ions in multiferroicity still remain unclear.

In this study, we investigated the magnetic and magnetoelectric features of a frustrated antiferromagnet, YCMO. We have first observed the emergence of ferroelectricity perpendicular to the *c* axis in a single crystalline YCMO. Isothermal magnetization along the *c* axis is observed to exhibit ferromagnetic-like hysteresis with large remanent magnetization, which is strongly correlated with the isothermal dielectric constant and ferroelectric polarization perpendicular to the *c* axis below *T*_N_ = 52 K. Based on the results of recent neutron diffraction experiments on YCMO, we proposed that the symmetric exchange striction between Yb^3+^ and Co^2+^/Mn^4+^ moments may lead to additional ferroelectricity along the *c* axis below *T*_Yb_ ≈ 20 K^44^. We note that in type-II multiferroics, multiple ferroelectricity, in which two different sorts of ferroelectricity emerge with magnetic origins, has scarcely been observed. However, the existence of additional ferroelectric polarization was not verified. Instead, dielectric anomaly along the *c* axis was observed. Our findings indicate distinct characteristics of the double-perovskite multiferroic and lay important groundwork for investigating materials with improved magnetoelectric functionalities.

## Results and discussion

**Figure 1:**
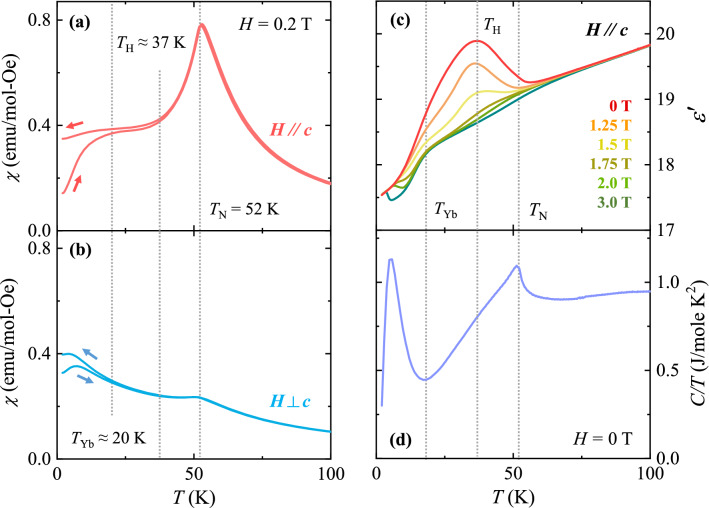
Temperature-dependent physical properties of Yb_2_CoMnO_6_ (YCMO). (**a**) and (**b**) Temperature dependence of magnetic susceptibility, *χ* = *M*/*H*, of the YCMO crystal at *H* = 0.2 T upon warming after zero-magnetic-field cooling and upon cooling in the same field along and perpendicular to the *c* axis, respectively. The vertical dotted lines represent the ordering temperature forming ↑↑↓↓-type spin state (*T*_N_ = 52 K), onset temperature of magnetic hysteresis (*T*_H_ ≈ 37 K), and ordering temperature of Yb^3+^ moments (*T*_Yb_ ≈ 20 K). (**c**) Temperature dependence of dielectric constant (*ε*′), measured perpendicular to the *c* axis at *H* = 0, 1.25, 1.5, 1.75, 2.0, 3.0 T along the *c* axis. (**d**) Temperature dependence of specific heat divided by the temperature, *C*/*T*, measured at *H* = 0 T.

YCMO crystallizes in a monoclinic *P*2_1_/*n* structure with lattice parameters *a* = 5.177 Å, *b* = 5.548 Å, and *c* = 7.418 Å with *β* = 89.648°^41^. The magnetic properties of YCMO were examined based on the temperature (*T*) dependence of magnetic susceptibility, defined as magnetization (*M*) divided by the magnetic field (*H*), *χ* = *M*/*H*, measured at *H* = 0.2 T on warming after zero-*H*-cooling (ZFC) and on cooling at the same *H* (FC). Figure 1a and b shows the anisotropic *χ* curves at *T* = 2–100 K for the two different orientations, *H*//*c* and *H* $\perp$ c, respectively. As *T* decreases from 100 K, both the ZFC and FC *χ* curves along the *c* axis increase smoothly with a nearly identical shape; they show a sharp anomaly at *T*_N_ = 52 K, which is considered to be the emergence of the ↑↑↓↓-type spin order^41,44^. Upon further cooling below *T*_N_, the *χ* curves decrease considerably and begin to split around *T*_H_ ≈ 37 K, which indicates the onset of thermally hysteretic behavior similar to that of Lu_2_CoMnO_6_^45^. Additional and much larger splitting occurs below *T*_Yb_ ≈ 20 K, where the Yb^3+^ moments are ordered. The *χ* values for *H*//*c* and *H* $\perp$ c indicate strong anisotropy, consistent with the spins primarily aligned along the *c* axis.

In Fig. 1c, the dielectric constant (*ε*′) perpendicular to the *c* axis, measured at *f* = 100 kHz and *H* = 0, 1.25, 1.5, 1.75, 2.0, and 3.0 T along the *c* axis, is plotted. At zero *H*, *ε*′ decreases almost linearly from 100 K and starts to increase near *T*_N_ = 52 K. It exhibits a broad peak at *T*_H_, followed by a slight slope change around *T*_Yb_. As *H* along the *c* axis is increased, the broad peak at *T*_H_ is progressively suppressed, and the slope change at *T*_Yb_ becomes clearer. The peak completely disappears at 3.0 T. The heat capacity divided by the temperature (*C*/*T*) also shows a sharp anomaly at *T*_N_, indicating a long-range order of the Co^2+^ and Mn^4+^ moments (Fig. 1d), consistent with that observed in Lu_2_CoMnO_6_^21^. An additional increase in *C*/*T* is found at *T*_Yb_, and a broad peak below *T*_Yb_ implies the long-range antiferromagnetic order of Yb^3+^ spins (Fig. 1d)^44^.

**Figure 2:**
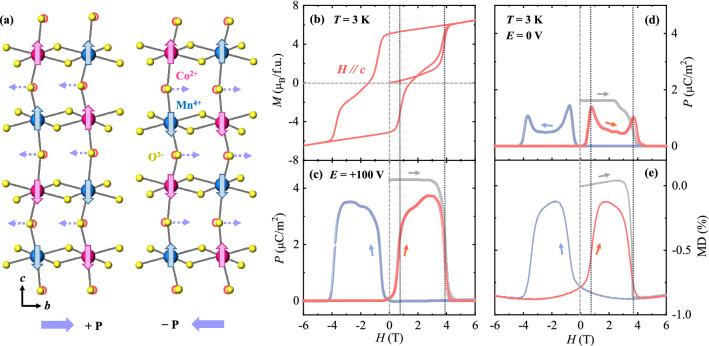
Origin of ferroelectricity in frustrated antiferromagnetic domains and magnetoelectrically hysteretic behavior. (**a**) Two types of frustrated antiferromagnetic domains. The purple dotted arrows denote the shifts in O^2−^ ions due to the exchange striction between parallel and antiparallel neighboring spins. (**b**) Isothermal magnetization of the YCMO crystal measured at *T* = 3 K in an *H* range of ± 6 T along the *c* axis. (**c**) and (**d**) Isothermal polarization perpendicular to the *c* axis at *T* = 3 K and *H* along the *c* axis, measured with *E* = 100 and 0 V, respectively. (**e**) Magnetodielectric (MD) effect defined as MD (%) = $\frac{{\varepsilon }^{^{\prime}}\left(H\right)-{\varepsilon }^{^{\prime}}(0 \mathrm{T})}{{\varepsilon }^{^{\prime}}(0 \mathrm{T})}\times 100$ perpendicular to the *c* axis for *H* up to ± 6 T along the *c* axis.

A previous neutron diffraction experiment on isostructural Lu_2_CoMnO_6_ established the magnetic structure of alternating Co^2+^ and Mn^4+^ moments as ↑↑↓↓-type spin configurations along the *c* axis^46^. The ↑↑↓↓ spin arrangement is driven by the nearest-neighbor ferromagnetic exchange interaction frustrated with comparable next-nearest-neighbor antiferromagnetic interactions. This system is called a frustrated antiferromagnet because the magnetic moments between ↑↑ and ↓↓ spin layers are canceled out, similar to an A-type antiferromagnet. A recent neutron diffraction study on YCMO has shown the same ↑↑↓↓-type arrangement of Co^2+^ and Mn^4+^ moments^41^. Figure 2a depicts the two types of frustrated antiferromagnetic domains of YCMO projected onto the *bc* plane. In the ↑↑↓↓ spin configuration as one type of domain, the parallel spin pairs of Co^2+^ and Mn^4+^ ions tend to contract, while the antiparallel spin pairs tend to expand, based on the exchange striction, which prefers ferromagnetic nearest-neighbor coupling. As a result, the contraction and expansion of the spin pairs distort between O^2−^ ions with cooperative displacements to the left and generate ferroelectric polarization (*P*) along the *b* axis, + *P*. For the other type of domains with the ↑↓↓↑ spin configuration, the exchange striction induces displacements of O^2−^ ions to the right and thus produces − *P*.

The full magnetic curve for *H*//*c* was recorded at 3 K after ZFC up to ± 6 T, as shown in Fig. 2b. The ↑↑↓↓-type spin order exhibits a ferromagnetic-like hysteresis loop with remanent *M*, *M*_r_ = 5.1 *μ*_B_/f.u., and magnetic coercive field, *H*_c_ = 1.4 T. The initial curve increases slowly and shows a sudden jump at ~ 3.8 T, indicating a change in the spin state from ↑↑↓↓ to ↑↑↑↑. Above this value, *M* increases linearly without saturation. The value of *M* at 6 T is found to be ~ 6.5 *μ*_B_/f.u., ascribed to the magnetic moments of Yb^3+^ ions in addition to the *M* value of 6 *μ*_B_ for the Co^2+^ (*S* = 3/2) and Mn^4+^ (*S* = 3/2) moments in a formula unit. Upon decreasing *H* from 6 T, *M* reduces progressively until it shows an abrupt decrease at approximately –0.8 T with the formation of the ↑↑↓↓-type magnetic domains. As *H* is further decreased, another step arises at –3.8 T for the change in the spin state to ↓↓↓↓. The antisymmetric shape of the loop is accomplished by sweeping *H* in the other direction.

The magnetoelectrically hysteretic behavior of YCMO was examined through the isothermal ferroelectric *P*, obtained by integrating the pyroelectric current density measured after poling in an electric field (*E* = 100 V or *E* = 2.3 kV/cm) perpendicular to the *c* axis and *H* up to ± 6 T along the *c* axis. In Fig. 2c, P was measured at 3 K by sweeping *H* without removing *E* after poling. The magnitude of *P* at 0 T was 4.3 μC/m^2^. *P* in the initial curve disappears at 3.8 T, consistent with the change in the spin state from ↑↑↓↓ to ↑↑↑↑. By decreasing *H* from 6 T, *P* is still zero until it shows a sudden jump at –0.8 T owing to the almost recovery of the ↑↑↓↓ state. *P* is reduced to zero again at –3.8 T for the change in the spin state to ↓↓↓↓. A similar hysteretic behavior was observed for the other directions of *H*. In Fig. 2d, P was measured by sweeping *H* at *E* = 0 V after poling. The *P* value at 0 T was reduced to 1.6 μC/m^2^, which suggests the incorporation of a considerable portion of the ↑↓↓↑-type antiferromagnetic domains with the opposite sign of *P*. The overall value of *P* was diminished, but a similar hysteretic behavior was detected. In addition, peak anomalies were observed when *P* was reduced to zero and recovered. In Fig. 2e, the magnetodielectric (MD) effect, described by the variation in *ε*′ by applying *H* and defined as MD (%) = $\frac{{\varepsilon }^{^{\prime}}\left(H\right)-{\varepsilon }^{^{\prime}}(0 \mathrm{T})}{{\varepsilon }^{^{\prime}}(0 \mathrm{T})}\times 100$, was measured at *f* = 100 kHz and *T* = 3 K perpendicular to the *c* axis up to ± 6 T along the *c* axis. *ε*′ reveals a hysteretic behavior similar to that of *P*, which reflects the ferroelectric domain motion.

**Figure 3:**
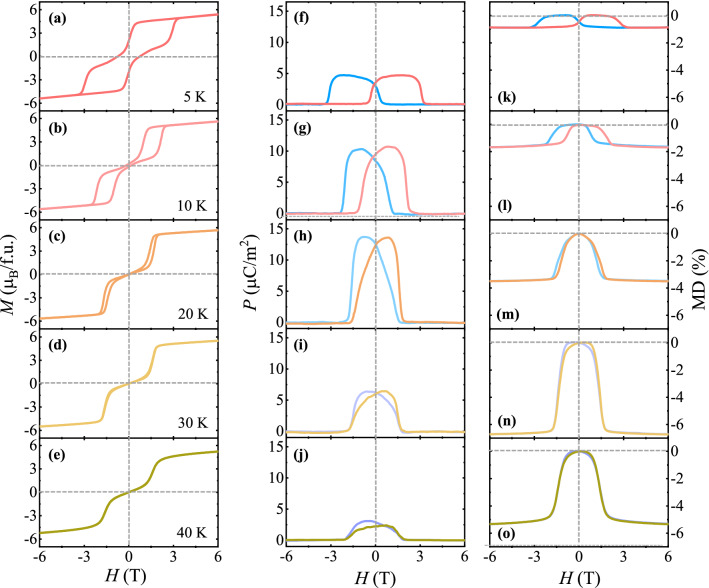
Temperature evolution of magnetic and ferroelectric properties. (**a**)–(**e**) Isothermal magnetization at *H* up to ± 6 T along the *c* axis and *T* = 5, 10, 20, 30, and 40 K. (**f**)–(**j**) Ferroelectric polarization perpendicular to the *c* axis at *H* up to ± 6 T along the *c* axis and *T* = 5, 10, 20, 30, and 40 K, obtained by integrating the magnetoelectric current measured and changing *H* at the rate of 0.01 T/s up to ± 6 T after poling at *E* = 2.3 kV/cm. (**k**)–(**o**) Magnetodielectric (MD) effect defined as MD (%) = $\frac{{\varepsilon }^{^{\prime}}\left(H\right)-{\varepsilon }^{^{\prime}}(0 \mathrm{T})}{{\varepsilon }^{^{\prime}}(0 \mathrm{T})}\times 100$ perpendicular to the *c* axis for *H* up to ± 6 T along the *c* axis at *T* = 5, 10, 20, 30, and 40 K.

**Figure 4:**
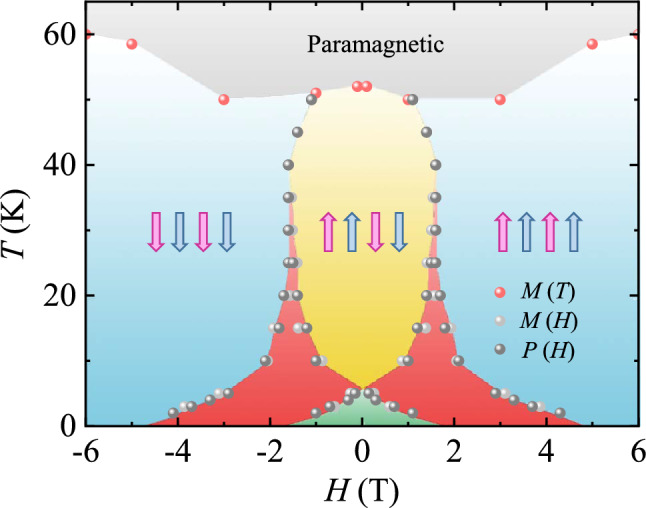
Phase diagram. The *H*-*T* phase diagram of the YCMO single crystal for *H* along the *c* axis, constructed from *T* dependence of *M*, and *H* dependence of *M* and *P*. In the red region, magnetic structure depends on the hysteresis.

To examine the behavior of full magnetoelectric hysteresis in detail, the *T* evolution of various physical properties was measured. Figure 3 shows a comparison among isothermal *P* (measured at *E* = 100 V) and MD effect perpendicular to the *c* axis, and *M* along the *c* axis at *H* up to ± 6 T and *T* = 5, 10, 20, 30, and 40 K. As *T* is increased, the hysteretic behavior is gradually suppressed. At 5 K, the area within the magnetic hysteresis loop is reduced (Fig. 3a), accompanied by a large reduction in *M*_r_ = 1.73 *μ*_B_/f.u. and *H*_c_ = 0.78 T, in comparison with *M* at 3 K (Fig. 2b). Owing to the narrowed hysteresis loop, double-step variations in *M* occur at *H* = 0.24 and –3.07 T upon sweeping *H* from 6 T. At 10 K, the magnetic hysteresis narrows further with *M*_r_ = 0.23 *μ*_B_/f.u. and *H*_c_ = 0.17 T (Fig. 3b). As *T* increases further, the area of the magnetic hysteresis loop of *M* rapidly shrinks, and *M*_*r*_ and *H*_*c*_ are nearly suppressed. At 40 K, the magnetic hysteresis in *M* almost disappears, while the double-step transitions remain (Fig. 3e). As shown in Fig. 3f, P at 5 K shows a hysteretic behavior similar to *M* following the appearance and disappearance of ferroelectricity at the double steps, which suggests a strong correlation between the magnetic and ferroelectric properties. As *T* increases further, the hysteretic behavior of *P* is reduced, but the magnitude of *P* is considerably enhanced up to 20 K (Fig. 3g and h). The maximum *P* values at 10 and 20 K were found to be 10.71 and 13.74 μC/m^2^, respectively. Above 20 K, the magnitude of *P* decreases with further reduction in the hysteretic behavior (Fig. 3i and j). The MD also tends to behave akin to *P*, which reflects the *H*-driven variations in ferroelectricity (Fig. 3k–o). The variation in MD is progressively enhanced as *T* increases. At 30 and 40 K, the maximum MD values were observed as − 6.68% and − 5.26%, respectively, at 6 T. These large variations arise at the *T* regime close to *T*_H_ ≈ 37 K, at which the broad peak of *ε*′ occurs (Fig. 1c). A recent X-ray photon correlation spectroscopy study on Lu_2_CoMnO_6_ clarified that the ↑↑↓↓-type arrangement, which is slightly incommensurate (ICM) with ***k*** = (0.0223(8), 0.0098(7), 0.5)^46^, emerges at *T*_N_ = 48 K and commensurate (CM) spin order corresponding to ***k*** = (0, 0, 0.5) arises below *T*_H_ = 30 K, while the ICM order still remains^47^. This suggests that the strong magnetic hysteresis originates from the simultaneous presence of the ICM and CM orders. The comprehensive behavior of strong magnetoelectric hysteresis in YCMO is demonstrated in the *H*-*T* phase diagram constructed from *T* dependence of *M*, and *H* dependence of *M* and *P*, as shown in Fig. 4. The data points for the phase boundaries were attained from the maximum slopes of *H* dependence of *M* and *P*. The onset of magnetoelectric hysteresis emerges at *T*_H_ ≈ 37 K, below which the hysteretic regime expands continuously. The red regime in which the corresponding magnetic state relies on magnetic hysteresis is clearly presented. The *P* is zero in the green colored region, corresponding to ↑↑↑↑ or ↓↓↓↓ Co^2+^/Mn^4+^ spin configuration due to the large magnetoelectric hysteresis at low *T* regime.

**Figure 5:**
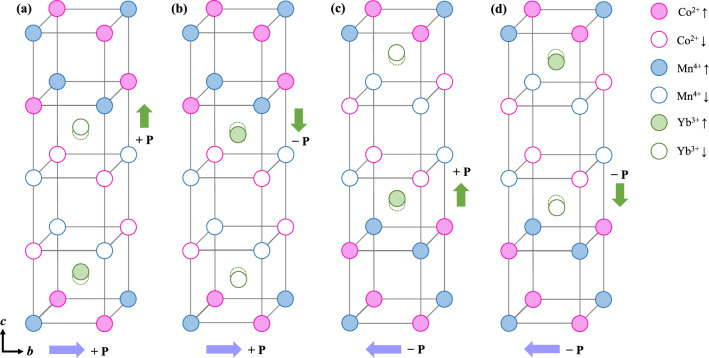
Proposed magnetoelectric domains of YCMO. Four types of magnetoelectric domains constructed from recent neutron diffraction experiments. The pink, blue, and green circles denote Co^2+^, Mn^4+^, and Yb^3+^ ions, respectively. The filled and open circles represent up and down spins, respectively, and the green dotted circle indicates the original position of the Yb^3+^ ions. In addition to *P* along the *b* axis, the exchange striction between Yb^3+^ and Co^2+^/Mn^4+^ magnetic moments induces *P* along the *c* axis. The four types of magnetoelectric domains are distinguished as (*P*_*b*_, *P*_*c*_) = (+, +), (+, −), (−, +), and (−, −).

In a recent neutron diffraction study on YCMO, in addition to the ↑↑↓↓-type arrangement of Co^2+^ and Mn^4+^ moments, the ordering of Yb^3+^ spins has been observed^41^. From the spin structure attained from this previously investigated neutron diffraction, we propose schematic spin configurations forming four types of magnetoelectric domains and possible activation of the symmetric exchange striction between Yb^3+^ and Co^2+^/ Mn^4+^ moments, which generates an additional ferroelectric *P* along the *c* axis, as shown in Fig. 5. All spin configurations are established within the magnetic *P*_*a*_2_1_ symmetry, consistent with that of Lu_2_CoMnO_6_. In such a symmetry, Yb^3+^ spins are distinguished as two different sites. For Yb1 sites, Yb^3+^ spins turn out to be strongly disordered because of the formation of strong internal fields from the neighboring ferromagnetic arrangement of Co^2+^/ Mn^4+^ spins between the upper and lower layers, which inhibits the antiferromagnetic coupling of Yb^3+^ spins^41^. By contrast, at the Yb2 sites, the cancelation of the internal fields from neighboring antiferromagnetic arrangement enables the Yb^3+^ spins to be ordered^41^. In Fig. 5a, the Yb^3+^ moments in the Yb2 sites are ordered antiferromagnetically aside from the ↑↑↓↓ spin arrangement of the Co^2+^ and Mn^4+^ ions; they also prefer to align antiferromagnetically to the neighboring Co^2+^/ Mn^4+^ moments. In such configuration, the down (↓) Yb^3+^ spin tends to shift to the upward direction and the up (↑) Yb^3+^ spin also shifts to the upward direction owing to the exchange striction that prefers the antiparallel alignment between Yb^3+^ and Co^2+^/Mn^4+^ spins. Thus, the net ferroelectric *P* is produced along the *c* axis. Similarly, oppositely aligned ferroelectric *P* occurs in the spin configuration, as shown in Fig. 5b. The other two types of magnetoelectric domains are depicted in Fig. 5c and d. Accompanied by the ↑↓↓↑ spin arrangement of the Co^2+^ and Mn^4+^ ions, the antiferromagnetic order of Yb^3+^ spins also leads to + *P* and − *P* along the *c* axis, respectively. As a result, four different types of magnetoelectric domains form depending on both *P* directions, i.e., (*P*_*b*_, *P*_*c*_) = (+ , +), (+ , −), (− , +), and (− , −).

**Figure 6:**
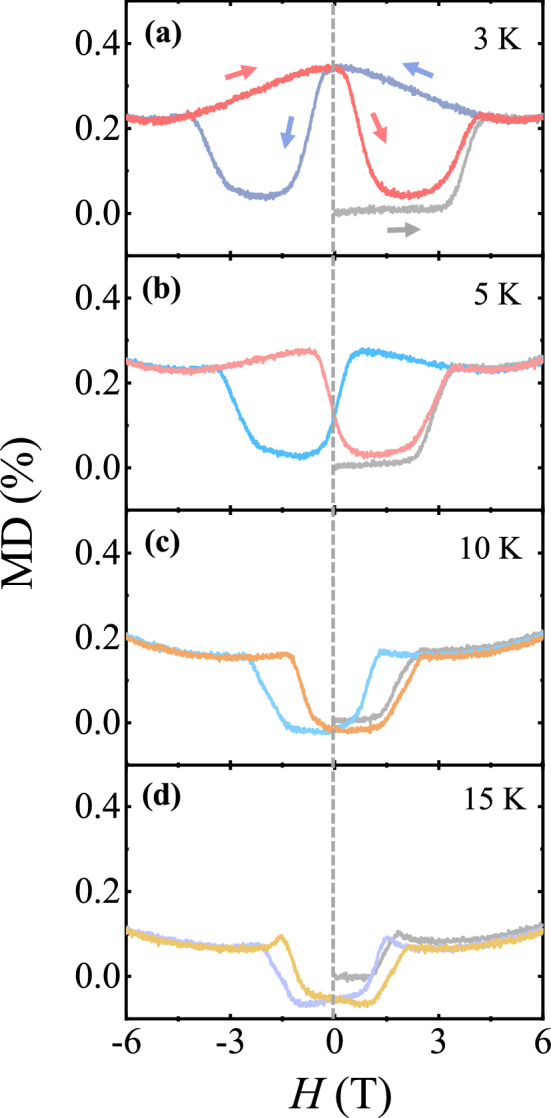
Temperature evolution of magnetodielectric effect along the *c* axis. (**a**)–(**d**) Magnetodielectric (MD) effect, MD (%) = $\frac{{\varepsilon }^{^{\prime}}\left(H\right)-{\varepsilon }^{^{\prime}}(0 \mathrm{T})}{{\varepsilon }^{^{\prime}}(0 \mathrm{T})}\times 100$, along the *c* axis for *H* up to ± 6 T along the *c* axis at *T* = 3, 5, 10, and 20 K.

To evaluate the potential occurrence of additional ferroelectricity along the *c* axis, we measured the electric properties. Contrary to the overall negative MD perpendicular to the *c* axis (Figs. 2e and 3k–o), the MD effect along the *c* axis with *H* up to ± 6 T along the *c* axis appears to be positive, and the magnitude of MD is largely reduced, as shown in Fig. 6. At 3 K, the initial curve exhibits a step-like jump in accordance with the spin-state variation from ↑↑↓↓ to ↑↑↑↑ (Fig. 6a). Following the magnetically hysteretic behavior, a rapid decrease in MD is observed at a negative value of *H*. A further decrease in *H* leads to a sudden jump, indicating the magnetic transition to the ↓↓↓↓ state. As *T* increases further, the magnitude of MD is continually reduced, which is different from the *T* evolution of the MD effect perpendicular to the *c* axis (Fig. 6b–d). Despite the observed positive MD effect with a strong dielectric anomaly, the presence of the proposed additional ferroelectricity has not been clearly proven because no measurable electric *P* was detected within the accuracy of our pyroelectric current measurement. Note that our measurement is highly sensitive to the *P* magnitude, as shown in the measured *P* perpendicular to the *c* axis (Figs. 2c, d, and 3f–j).

## Conclusion

In summary, we explored the anisotropic magnetoelectric properties of the frustrated antiferromagnet YCMO. Ferroelectric polarization was found to occur perpendicular to the *c* axis, originating from the exchange strictive shifts of O^2−^ ions of the ↑↑↓↓-type Co^2+^/Mn^4+^ spin order along the *c* axis. The magnetoelectric hysteresis observed below *T*_H_ ≈ 37 K revealed an interesting correlation between the magnetic and ferroelectric properties. We also proposed that the magnetic order of Yb^3+^ moments below *T*_Yb_ ≈ 20 K could lead to an additional ferroelectric polarization along the *c* axis with the formation of four different types of magnetoelectric domains. Our findings provide insights into fundamental magnetic and magnetoelectric interactions in the frustrated antiferromagnets of the double-perovskite family, inspiring the discovery of new compounds for functional magnetoelectric applications.

## Methods

Single crystals of double-perovskite YCMO were synthesized by the conventional flux method using Bi_2_O_3_ as a flux^40^. A polycrystalline specimen was first prepared by a solid-state reaction. High-purity powders of Yb_2_O_3_, Co_3_O_4_, and MnO_2_ were mixed in a stoichiometric ratio and ground in a mortar, followed by pelletizing and calcining at 1000 °C for 12 h in a furnace. The calcined pellets were reground and sintered at 1100 °C for 24 h. The same sintering procedure was repeated at 1200 °C for 48 h. A mixture of polycrystalline powder and Bi_2_O_3_ flux in a 1:12 ratio was heated to 1300 °C and melted in a Pt crucible. It was then slowly cooled to 850 °C at a rate of 1.5 °C/h and then cooled further to room temperature while the furnace was turned off.

The temperature and magnetic-field dependences of the DC magnetizations were measured using a vibrating sample magnetometer (VSM) at *T* = 2–100 K and *H* =  − 9–9 T in a physical properties measurement system (PPMS, Quantum Design, Inc.). The specific heat was measured using the standard relaxation method in the PPMS. The temperature and magnetic-field dependences of the dielectric constant were measured at *f* = 100 kHz using an LCR meter (E4980, Agilent). The temperature and magnetic-field dependences of electric polarization were obtained by integrating pyro- and magneto-electric currents, respectively, measured after poling in a static electric field, *E* = 2.3 kV/cm.
